# LYVE-1 identifies asthma and drives PDGF-BB-induced proliferation, migration, and oxidative stress in airway smooth muscle cells via the PI3K/Akt pathway

**DOI:** 10.3389/fphar.2026.1738301

**Published:** 2026-02-11

**Authors:** Yunbo Tang, Nan Zhou, Xiaohui Ni

**Affiliations:** Department of Respiratory and Critical Care Medicine, Nantong Second People’s Hospital, Nantong, Jiangsu, China

**Keywords:** airway inflammation, airway remodeling, asthma, PI3K/Akt pathway, sLYVE-1

## Abstract

**Background:**

Asthma is a chronic inflammatory airway disease characterized by airflow limitations, airway remodeling, and immune dysregulation. Lymphatic vessel endothelial hyaluronan receptor-1 (LYVE-1) has recently been implicated in inflammatory and remodeling processes in the body. However, its clinical significance and mechanistic role in asthma are unclear. This study aimed to investigate the association between soluble LYVE-1 (sLYVE-1) levels and asthma severity, airway inflammation, and remodeling, and to elucidate its regulatory role in airway smooth muscle cell (ASMC) activation via the PI3K/Akt signaling pathway.

**Methods:**

A total of 238 participants were enrolled, including 80 healthy controls, 72 patients with asthma who were in remission, and 86 patients with acute asthma. Clinical characteristics, pulmonary function parameters, inflammatory and remodeling biomarkers, and serum sLYVE-1 levels were assessed. Correlation analyses were performed to evaluate the relationship between sLYVE-1 and disease-related parameters. Mechanistic studies were conducted *in vitro* using platelet-derived growth factor-BB (PDGF-BB)-stimulated ASMCs with LYVE-1 knockdown to explore the downstream signaling and functional effects.

**Results:**

Serum sLYVE-1 levels were significantly elevated in patients with asthma and progressively increased with disease severity. sLYVE-1 levels were inversely correlated with forced expiratory volume in one second (FEV1) and FEV1/FVC and positively correlated with total immune globulin E (IgE), eosinophil counts, T-helper cell type 2 (Th2) and T-helper cell type 17 (Th17) cell proportions, fractional exhaled nitric oxide (FeNO), type 2 cytokines, and airway remodeling-associated mediators, including vascular endothelial growth factor A (VEGF-A), stromal-derived factor 1α (SDF-1α), transforming growth factor-β1 (TGF-β1), matrix metalloproteinases-9 (MMP-9), and hyaluronan (all *p* < 0.001). *In vitro*, LYVE-1 silencing markedly attenuated PDGF-BB-induced PI3K/Akt phosphorylation, ASMC proliferation and migration, extracellular matrix-related gene expression, and pro-inflammatory cytokine secretion while reducing oxidative stress and enhancing antioxidant activity.

**Conclusion:**

Elevated circulating sLYVE-1 levels are closely associated with asthma severity, airway inflammation, and airway remodeling. Mechanistically, LYVE-1 promoted PDGF-BB-induced ASMC activation through PI3K/Akt signaling, highlighting LYVE-1 as a potential biomarker and potential therapeutic target for asthma.

## 1 Introduction

Asthma is a prevalent and debilitating chronic respiratory disease characterized by airway hyperresponsiveness (AHR), chronic inflammation, and structural alterations in the airway wall, collectively known as airway remodeling (Venkatesan, 2023; Lambrecht and Hammad, 2015). A central component of this remodeling process is airway smooth muscle (ASM) dysfunction, which involves profound phenotypic changes, including excessive proliferation, migration, and hypertrophy (Bentley and Hershenson, 2008). These aberrant ASM behaviors contribute directly to increased airway wall thickness, heightened contractility, and a decline in lung function, underscoring the critical need to elucidate the molecular drivers of ASM pathobiology (Prakash, 2013).

Platelet-derived growth factor (PDGF), particularly the BB homodimer (PDGF-BB), is a potent mitogen and chemoattractant for ASM cells and is strongly implicated in the asthmatic airway remodeling cascade (Heldin and Lennartsson, 2013). PDGF-BB signaling through its receptor PDGF-Rβ activates multiple downstream pathways, with the phosphoinositide 3-kinase (PI3K)/Akt axis being the principal mediator of cellular survival, growth, and metabolic stress (Ebenezer et al., 2016). The PI3K/Akt pathway regulates key processes in ASM, such as proliferation and generation of reactive oxygen species (ROS), which further amplify inflammatory and remodeling signals (Yu et al., 2016; Song et al., 2025). However, the precise regulatory mechanisms and upstream modulators that govern the PDGF-BB/PI3K/Akt axis in asthma remain unclear.

Recent evidence indicates that PDGF/PDGFR signaling can be modulated by non-coding RNAs, including microRNAs, long non-coding RNAs (lncRNAs), and circular RNAs (circRNAs), suggesting novel therapeutic opportunities for diseases involving smooth muscle remodeling (Ma et al., 2024). Phenotypic switching of smooth muscle cells, driven by growth factor signaling, has been recognized as a key mechanism in vascular and airway remodeling, contributing to disease progression (Chigareva et al., 2025). In addition, other growth factors, including VEGF and TGF-β, act in concert with PDGF to promote vascular and airway remodeling, highlighting the complex interplay between proliferative and inflammatory signaling in asthma (Yamamura, 2025).

Lymphatic vessel endothelial hyaluronan receptor-1 (LYVE-1) is a transmembrane glycoprotein that serve as a specific marker for lymphatic endothelial cells (Bano et al., 2025). While its classical role involves hyaluronan metabolism and lymphatic function, emerging evidence suggests that LYVE-1 expression may not be restricted to the endothelium. Notably, its involvement in other fibroproliferative diseases suggests a potential role in cellular activation and tissue remodeling (Gordon et al., 2008; Lim et al., 2018). Despite this expanding biological relevance, there are currently no published human airway transcriptomic studies demonstrating the significant upregulation of LYVE1 in airway tissues from patients with asthma. In contrast, transcriptomic analyses of asthmatic airways have consistently identified other genes, including CST1, POSTN, SERPINB2, and inflammatory mediators, as significantly upregulated in the bronchial and nasal epithelium, highlighting the robustness of airway transcriptomics in defining asthma-associated molecular signatures (Wang et al., 2023). Consequently, the functional significance of LYVE-1 in airway smooth muscle (ASM) biology and its potential interaction with established pro-remodeling pathways, such as PDGF-BB/PI3K/Akt signaling, remain undefined and warrant further investigation.

In this study, we investigated the hypothesis that LYVE-1 not only serves as a biomarker for asthma but also actively promotes PDGF-BB-induced ASMC pathological behaviors, specifically proliferation, migration, and oxidative stress, via activation of the PI3K/Akt signaling pathway. By elucidating the mechanistic involvement of LYVE-1 in ASMC dysfunction and airway remodeling, this study aimed to identify a novel therapeutic axis for mitigating structural airway changes in asthma.

## 2 Patients and methods

### 2.1 Study population

**FIGURE 1:**
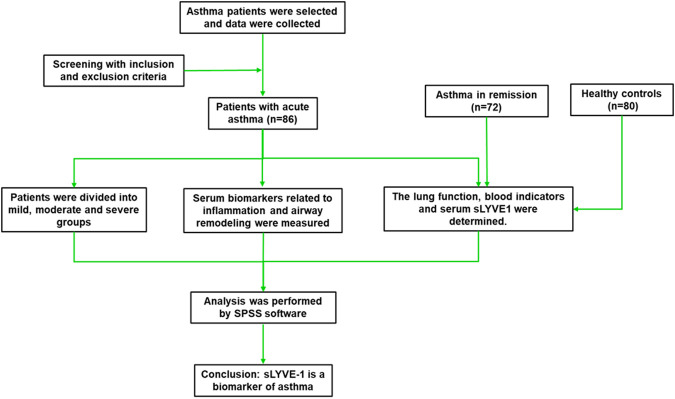
Schematic overview of the study design and experimental workflow.

A total of 86 patients with acute asthma from the Respiratory Department in our Hospital were enrolled between January 2021 and December 2024. Asthma diagnosis was based on the diagnostic criteria of the Global Initiative for Asthma (GINA) (Girodet and Molimard, 2021). Asthma severity was assessed and further divided into mild (n = 41), moderate (n = 26), and severe (n = 19) groups. The inclusion criteria were as follows: (i) in line with the diagnostic criteria of the Global Initiative for Asthma; (ii) age ≥18 years; (iii) first onset of illness; and (iv) complete clinical data. The exclusion criteria were as follows: (i) other respiratory or immune system-related diseases; (ii) diseases that combine with circulatory, digestive, urinary, and neurological systems; (iii) history of allergic diseases or family history of asthma; and (iv) use of medications such as glucocorticoids within the past 3 months. The control group included healthy subjects who came for routine health screenings or outpatient asthma patients on admission with matched age and sex. The research procedure complied with the Declaration of Helsinki and was approved by the Medical Ethics Committee of our hospital. Figure 1 illustrates the overall framework of this study.

### 2.2 Clinical data collection

The complete clinical and laboratory data of enrolled patients with asthma in remission and with acute asthma were collected, including age, sex, body mass index (BMI), smoking status, total immune globulin E (IgE), blood eosinophils, and blood neutrophils. Pulmonary function tests (PFT) were performed using portable spirometry to determine forced expiratory volume in one second (FEV1) and forced vital capacity (FVC). The FEV1/FVC ratio was FEV1 predicted was calculated according to the following formula: male: 0.041 × height (cm) - (0.018 × age) - 2.69; female: 0.033 × height (cm) - (0.022 × age) - 2.49.

### 2.3 Serum collection

Whole blood (5 mL) was collected from fasting participants between 8:00 a.m. and 9:00 a.m. using serum separation tubes without anticoagulant. The samples were allowed to clot at room temperature for 30 min and were then centrifuged within 4 h at 3,000 rpm for 10 min. The supernatant serum was carefully collected, aliquoted to avoid repeated freeze-thaw cycles, and stored at −80 °C until further analysis.

### 2.4 Isolation of peripheral blood mononuclear cells (PBMCs)

Whole blood (5 mL) was collected from each fasting participant and placed in a common biochemical tube. This blood sample was mixed with equal volume phosphate buffered saline (PBS) to form a diluent of peripheral blood. This diluted peripheral blood was added slowly in another centrifuge tube containing lymphocyte separation solution (Ficoll-Paque™ PLUS; GE Healthcare, Chicago, IL, United States; Cat. No. 17–1440–03). Then the solution was centrifuged at room temperature for 20 min (2000 r/min), and obvious delamination can be seen in the centrifuge tube. The white film layer was drawn with a Pasteur tube, and place it in a new centrifuge tube. After washed by PBS, the supernatant was discarded and cell pellet was obtained as PBMCs and counted.

### 2.5 Flow cytometry (Th2 cells and Th17 cells)

After isolation of PBMCs, 1000 μL of RPMI 1640 medium was added to resuspend the cell pellet, followed by centrifugation at 300 *g* for 5 min, the supernatant was discarded, and the cells were resuspended with 100 μL of pre-cooled PBS. The PBMCs were incubated with anti-human CD3 mAb (BD Biosciences, San Jose, CA, United States; Cat. No. 555339) and anti-human CD8 mAb (BD Biosciences; Cat. No. 555369) at 4 °C in the dark for 30 min (for anti-labeling CD4 + T cells). Then cells were washed by 3 mL pre-cooled PBS by centrifugation at 300 *g* for 5 min. After discarding the supernatant, cells were resuspend with PBS. Cells were incubate with 100 μL membrane permeabilization solution (BD Cytofix/Cytoperm™, BD Biosciences; Cat. No. 554714) at room temperature for 10 min, and then add with anti-human IL-4 mAb (BD Biosciences; Cat. No. 554486) and anti-human-IL-17A mAb (BD Biosciences; Cat. No. 560486), respectively. After incubation at 4 °C in the dark for 30 min, cells were washed with PBS, and resuspend by adding 300 μL pre-cooled PBS and immediately perform a BD FACSCanto™ II flow cytometer (BD Biosciences) to analyze Th2 and Th17 cells. Data acquisition and analysis were performed using FlowJo software (version 10.8.1; BD Biosciences). (CD3^+^ CD8^−^IL-IL-4^+^) accounted for the proportion of Th2 cell population, and (CD3^+^CD8^−^IL-17A^+^) accounted for the proportion of Th17 cell population.

### 2.6 Fractional exhaled nitric oxide (FeNO)

FeNO levels were measured using a nitric oxide analyzer (Sunvou-CA2122 system, China) that operates at a 50 mL/s exhalation flow rate, with the results expressed in parts per billion (ppb) (Menzies et al., 2007). Fractional exhaled nitric oxide (FeNO) and nasal nitric oxide (FnNO) levels were measured. Each participant underwent two measurements, maintaining an exhalation time of 10 s for each test.

### 2.7 Measurement of serum biomarkers

An enzyme-linked immunosorbent assay (ELISA) was used to measure the serum levels of interleukin-4 (IL-4; D4050, R&D Systems), interleukin-5 (IL-5; D5000B, R&D Systems), interleukin-13 (IL-13; D1300B, R&D Systems), vascular endothelial growth factor A (VEGF-A; SEKH-0052, Solarbio), stromal cell-derived factor 1 alpha (SDF-1α; ml038196, Shanghai Enzyme-linked Biotechnology), transforming growth factor beta 1 (TGF-β1; DY240, R&D Systems), matrix metallopeptidase 9 (MMP-9; DMP900, R&D Systems), hyaluronan (DHYAL0, R&D Systems), and soluble lymphatic vessel endothelial hyaluronan receptor 1 (sLYVE1; DY2089, R&D). Experiments were performed according to the manufacturer’s instructions.

### 2.8 Cell culture

Human airway smooth muscle cells (ASMCs) were purchased from ScienCell Research Labs (Carlsbad, CA, USA) and cultured in high-glucose DMEM (Gibco, Waltham, MA, USA) and 10% FBS (Gibco) at 37 °C with 5% CO_2_. ASMCs were divided into four groups: (i) Control group: conventional culture; (ii) Model group (PDGF-BB + si-NC): cells were transfected with si-NC for 24 h, and then treated with PDGF-BB (20 ng/mL) for further 24 h; (iii) LYVE-1 silencing group (PDGF-BB + si-LYVE-1): cells were transfected with si-LYVE-1 for 24 h, and stimulated with 20 ng/mL PDGF-BB for 24 h (iv) Pathway inhibitor group (PDGF-BB + si-LYVE-1 + IGF-1): Cells were transfected with si-LYVE-1 for 24 h and then co-treated with IGF-1 (100 ng/mL) and PDGF-BB (20 ng/mL) for a further 24 h.

### 2.9 Cell transfection

ASMCs in the logarithmic growth phase were cultured in high-glucose DMEM with 10% FBS and seeded in 6-well plates (1.0 × 10^5^ cells/well). Cells were starved in DMEM with 1% FBS for 12 h. LYVE-1 siRNA (si-LYVE-1) or its negative control (si-NC) was introduced into ASMCs using Lipofectamine 2000 (Invitrogen, Carlsbad, CA, USA). After incubation overnight, the cells were replaced with DMEM with 10% FBS. After 48 h, transfection efficiency was verified using RT-qPCR and Western blotting. LYVE-1 siRNA and control siRNA sequences were synthesized by Suzhou Jima Gene Co., Ltd. The sequence of siRNA is as follows: si-LYVE-1-1: (forward, 5′GCA GCA GCA UGA AGA AAC A-3′; reverse, 5′-CGU CGU CGU ACU UCU UUG U-3′), si-LYVE-1-2: (forward, 5′-GCU GCU GCU UUG GAU GAA G-3′; reverse, 5′-CGA CGA CGA AAC CUA CUU C-3′); si-LYVE-1-3: (forward, 5′-CCA UGU GGA UGC UGA UGA A-3′; reverse, 5′-GGU ACA CCU ACG ACU ACU U-3′); si-NC (forward, 5′-UUC UCC GAA CGU GUC ACG U-3′; reverse, 5′-AAG AGG CUU GCA CAG UGC A-3′).

### 2.10 MTT assay

Cell viability was assessed using the MTT assay. Airway smooth muscle cells (ASMCs) were seeded in 96-well plates at a density of 1 × 10^4^ cells/well and cultured overnight at 37 °C in a humidified atmosphere containing 5% CO_2_ to allow cell attachment. After the indicated treatments, 20 µL of MTT solution (1 mg/mL; M1020, Solarbio, Beijing, China) was added to each well and incubated for 3–4 h at 37 °C to allow the formation of purple formazan crystals. The supernatant was carefully removed, and 100 µL of dimethyl sulfoxide (DMSO) was added to dissolve the formazan crystals. Absorbance was measured at 570 nm using a microplate reader, and the background absorbance from the blank wells was subtracted. Cell viability was expressed as a percentage of the untreated control according to the following formula: viability (%) = [(A_sample-A_blank)/(A_control-A_blank)] × 100. All experiments were performed in at least six technical replicates and repeated independently thrice to ensure reproducibility.

### 2.11 Cell migration assay

Cell migration was evaluated using a Transwell chamber assay. Cell migration was assessed using 24-well Transwell inserts (BD Biosciences, San Diego, CA, USA) with 8-μm pore polycarbonate membranes. ASMCs were resuspended in serum-free medium, and 5 × 10^4^ cells in 500 μL were seeded into the upper chamber of each insert. The lower chamber was filled with 700 μL of complete medium containing 10% FBS, which served as a chemoattractant. After incubation for the indicated time at 37 °C in a humidified 5% CO_2_ atmosphere, non-migrated cells remaining on the upper surface of the membrane were gently removed using a cotton swab. The migrated cells on the lower surface were fixed with 4% paraformaldehyde for 15 min and stained with 0.1% crystal violet for 10–15 min at room temperature. The stained cells were washed with PBS to remove excess dye, air-dried, and imaged under an inverted microscope at ×100 magnification. The migrated cells were counted in at least five randomly selected microscopic fields per insert, and the average number was used for the statistical analysis. All experiments were performed in triplicate to ensure reproducibility.

### 2.12 ELISA

Culture supernatants from treated ASMCs were collected, centrifuged at 1,000–2,000 × g for 5 min to remove cellular debris, aliquoted, and stored at −80 °C until analysis. Secreted levels of TNF-α (DTA00D), IL-1β (DLB50), and IL-6 (D6050B) were quantified using commercial enzyme-linked immunosorbent assay (ELISA) kits (R&D Systems) according to the manufacturer protocol. Briefly, standards and samples were loaded in duplicate onto pre-coated 96-well plates, incubated with the supplied detection reagents, and subjected to the recommended wash steps to remove unbound antibodies. After the addition of the substrate solution and termination of the reaction, the optical density was measured at 450 nm with a wavelength correction of 540–570 nm using a microplate reader.

### 2.13 Intracellular ROS level

Intracellular reactive oxygen species (ROS) levels were measured using the fluorescent probe dihydroethidium (DHE; S0063, Beyotime). Briefly, after the indicated treatments, ASMCs cultured on glass-bottom dishes or coverslips were washed twice with warm PBS and incubated with DHE (10 µM in serum-free medium) for 30 min at 37 °C in the dark. Following incubation, the cells were gently washed twice with PBS and immediately imaged live using an inverted fluorescence microscope at ×200 magnification. DHE-derived red fluorescence (oxidized ethidium intercalated into DNA) was detected using the appropriate red fluorescence filter settings. For each condition, images were acquired from at least five randomly selected fields per replicate, and experiments were performed in three independent biological replicates for each condition. Intracellular ROS levels were quantified by measuring the mean fluorescence intensity per field using ImageJ (NIH) after background subtraction and normalization to the cell number.

### 2.14 Oxidative stress

Oxidative stress in ASMCs was evaluated by determining the levels of malondialdehyde (MDA), superoxide dismutase (SOD), and glutathione peroxidase (GPx) using commercial assay kits (Beyotime, Shanghai, China). Lipid peroxidation was quantified by measuring the MDA concentration using an MDA assay kit (S0131S), and absorbance was measured at 532 nm. SOD activity was determined using an SOD assay kit (S0109), and the absorbance was measured at 450 nm. GPx activity was assessed using a GPx assay kit (S0056) according to the manufacturer’s instructions, and the absorbance was measured at 340 nm. All measurements were performed in triplicate and expressed as units per milligram of protein or relative to control values. Each experiment was independently repeated at least thrice to ensure reproducibility.

### 2.15 RT-qPCR

Total RNA was extracted from the treated airway smooth muscle cells using TRIzol reagent (Invitrogen) according to the manufacturer’s instructions, and RNA concentration and purity (A260/A280) were assessed using a NanoDrop spectrophotometer. One microgram of total RNA was reverse-transcribed to cDNA using a commercial reverse transcription kit (PrimeScript RT kit, Takara) in a 20 µL reaction, following the manufacturer’s protocol. Quantitative real-time PCR (RT-qPCR) was performed on an ABI 7500 Real-Time PCR System (Applied Biosystems) using an SYBR Green–based master mix (SYBR Green PCR Master Mix, Applied Biosystems) in 20 µL reaction volumes containing 2 µL of cDNA and 400 nM of each primer. Primer sequences are listed in Supplementary Table S1 and the housekeeping gene used for normalization is indicated therein. The thermal cycling conditions were as follows: initial denaturation at 95 °C for 3 min, followed by 40 cycles of 95 °C for 10 s and 60 °C for 30 s, with a final melting curve analysis to verify product specificity. Each sample was run in technical triplicate, and the experiments were repeated at least three times independently. Relative transcript levels were calculated using the 2^−ΔΔCt^ method and expressed as fold changes relative to those of the control group.

### 2.16 Western blot

Protein expression was analyzed using Western blotting. After the indicated treatments, ASMCs were washed twice with ice-cold PBS and lysed in 100 µL RIPA buffer (Beyotime, Shanghai, China) supplemented with 1 µL of protease and phosphatase inhibitor cocktails (Roche). The lysates were incubated on ice for 30 min and centrifuged at 12,000 × g for 15 min at 4 °C to remove debris. Supernatants were collected for protein quantification using the bicinchoninic acid (BCA) assay (Thermo Fisher Scientific). Equal amounts of protein (50 µg) were separated by 10% SDS-PAGE and transferred onto polyvinylidene difluoride (PVDF) membranes (Millipore). After blocking with 5% nonfat milk in Tris-buffered saline containing 0.1% Tween-20 (TBST) for 1 h at room temperature, membranes were incubated overnight at 4 °C with the following primary antibodies: LYVE-1 (1:500, ab219556, rabbit monoclonal, Abcam), p-PI3K (p85, Tyr458; 1:400, ab278545, rabbit monoclonal, Abcam), PI3K (1:500, ab302958, rabbit monoclonal, Abcam), p-Akt (Ser473; 1:400, ab81283, rabbit monoclonal, Abcam), Akt (1:400, ab8805, rabbit polyclonal, Abcam), and GAPDH (1:2000, ab8245, mouse monoclonal, Abcam). Membranes were then washed with TBST and incubated with HRP-conjugated secondary antibodies for 1 h at room temperature. Protein bands were visualized using enhanced chemiluminescence (ECL) reagents (Thermo Fisher Scientific) and imaged using a ChemiDoc MP system (Bio-Rad). Densitometric analysis was performed using ImageJ software (NIH), and the protein expression levels were normalized to GAPDH and expressed relative to those of the control group. All experiments were independently repeated at least thrice.

### 2.17 Statistical analysis

All statistical analyses were performed using IBM SPSS Statistics software (version 20.0). Continuous data are expressed as mean ± standard deviation (SD). Categorical data were expressed as frequencies (percentages). Dermographic data were compared using one-way analysis of variance (ANOVA) or the Kruskal–Wallis test, followed by the LSD-Mean Multiple Comparison Test. Sex and smoking status were compared using the chi-square test. Pearson’s correlation analysis was performed to assess the correlations between sLYVE-1 levels, lung function, and other serum biomarkers. *p* < 0.05 was considered statistically significant.

## 3 Results

### 3.1 Clinical characteristics of study participants and disease severity

A total of 238 participants were included in this study: 80 healthy controls, 72 patients with asthma in remission, and 86 patients with acute asthma. There were no significant differences in age or sex distribution among the groups, while the body mass index was slightly lower in patients with acute asthma (22.06 ± 2.75 kg/m^2^) than in controls (23.55 ± 2.47 kg/m^2^) and patients with remission (23.12 ± 2.80 kg/m^2^; *p* = 0.001). The prevalence of smoking was comparable across the groups (32.5%, 38.9%, and 40.7%; *p* = 0.525). Pulmonary function was markedly impaired in acute asthma, with reductions in FEV1 (1.89 ± 0.56 L), FEV1/FVC ratio (65.58% ± 4.90%), and predicted FEV1 (58.74% ± 8.35%) compared to those in the controls (3.15 ± 0.72 L, 80.52% ± 5.23%, and 98.24% ± 13.47%, respectively; all *p* < 0.001). Serum total IgE (199.71 ± 57.65 IU/mL) and eosinophil percentage (3.66% ± 0.80%) were significantly elevated in acute asthma relative to controls and remission patients (both *p* < 0.001), whereas neutrophil percentages did not differ significantly (*p* = 0.140). The proportions of Th2 and Th17 cells were significantly higher in acute asthma (3.01% ± 0.63% and 2.62% ± 0.56%, respectively; *p* < 0.001), consistent with elevated airway inflammation, as indicated by FeNO levels (44.66 ± 7.20 ppb; *p* < 0.001). Importantly, serum soluble LYVE-1 (sLYVE-1) levels progressively increased from healthy controls (376.89 ± 55.38 ng/mL) to remission (462.27 ± 58.52 ng/mL) and acute asthma patients (593.94 ± 101.94 ng/mL; *p* < 0.001) (Table 1), suggesting a potential role for LYVE-1 as a biomarker associated with asthma severity and airway inflammatory status.

**TABLE 1:** Characteristics of the healthy controls and asthma patients.

Variables	Healthy controls (n = 80)	Asthma remission (n = 72)	Acute asthma (n = 86)	p-value
Age (years)	58.86 ± 9.95	57.18 ± 9.19	58.57 ± 9.14	0.507
Sex (male, %)	45 (56.3%)	40 (55.6%)	50 (58.1%)	0.943
BMI (kg/m^2^)	23.55 ± 2.47	23.12 ± 2.80	22.06 ± 2.75	0.001
Smoking (%)	26 (32.5%)	28 (38.9%)	35 (40.7%)	0.525
FEV1 (L)	3.15 ± 0.72	2.82 ± 0.79	1.89 ± 0.56	<0.001
FEV1/FVC (%)	80.52 ± 5.23	76.67 ± 5.50	65.58 ± 4.90	<0.001
FEV1 predicted (%)	98.24 ± 13.47	92.48 ± 11.47	58.74 ± 8.35	<0.001
Total IgE (IU/mL)	116.34 ± 34.49	129.31 ± 34.21	199.71 ± 57.65	<0.001
Eosinophil rate (%)	1.83 ± 0.62	1.98 ± 0.64	3.66 ± 0.80	<0.001
Neutrophil rate (%)	58.09 ± 9.24	59.09 ± 10.79	61.17 ± 10.57	0.140
Th2 (%)	1.04 ± 0.22	1.16 ± 0.27	3.01 ± 0.63	<0.001
Th17 (%)	0.97 ± 0.16	1.13 ± 0.20	2.62 ± 0.56	<0.001
FeNO (ppb)	15.22 ± 2.19	23.07 ± 2.90	44.66 ± 7.20	<0.001
SerumsLYVE1 (ng/mL)	376.89 ± 55.38	462.27 ± 58.52	593.94 ± 101.94	<0.001

Continuous variables are expressed as mean ± standard deviation (SD), and categorical variables are expressed as frequency (percentage). Comparisons between three groups were determined by chi-square test, one-way analysis of variance (ANOVA) or Kruskal–Wallis test followed by LSD-Mean Multiple Comparison Test. P < 0.05 was considered statistically significant.

Abbreviation: BMI, body mass index; FEV1, forced expiratory volume in one second; FVC, forced vital capacity; IgE, immune globulin E; Th2, T-helper cell type 2; Th17, T-helper cell type 17; FeNO, exhaled nitric oxide; sLYVE1, soluble lymphatic vessel endothelial hyaluronan receptor 1.

Among the 86 patients with asthma stratified by disease severity, 41 had mild asthma, 26 had moderate asthma, and 19 had severe asthma. There were no significant differences in age, sex distribution, BMI, or smoking prevalence across the severity groups. Pulmonary function declined progressively with increasing asthma severity, as evidenced by reductions in FEV1 (2.12 ± 0.54 vs. 1.76 ± 0.50 vs. 1.55 ± 0.46 L), FEV1/FVC ratio (67.73% ± 4.78% vs. 64.62% ± 4.17% vs. 62.25% ± 3.88%), and predicted FEV1 (62.42% ± 7.52% vs. 57.77% ± 7.30% vs. 52.15% ± 7.15%; all *p* < 0.001). Total serum IgE (179.27 ± 48.96 vs. 206.78 ± 55.15 vs. 234.15 ± 62.21 IU/mL; P = 0.002) and eosinophil percentages (3.27% ± 0.65% vs. 3.81% ± 0.71% vs. 4.33% ± 0.77%; *p* < 0.001) increased with severity, whereas neutrophil percentages remained similar (*p* = 0.560). The proportions of Th2 and Th17 cells were significantly elevated in moderate and severe asthma (Th2:2.69% ± 0.50% vs. 3.12% ± 0.53% vs. 3.54% ± 0.65%; Th17:2.28% ± 0.37% vs. 2.71% ± 0.43% vs. 3.25% ± 0.47%; both *p* < 0.001), consistent with increased airway inflammation, as reflected by FeNO levels (40.44 ± 5.01 vs. 45.67 ± 5.82 vs. 52.40 ± 6.08 ppb; *p* < 0.001). Similarly, type 2 cytokines IL-4, IL-5, and IL-13 increased progressively with disease severity (IL-4: 28.69 ± 3.92 vs. 33.36 ± 4.35 vs. 38.37 ± 4.91 pg/mL; IL-5: 83.31 ± 11.66 vs. 93.23 ± 12.69 vs. 101.08 ± 15.82 pg/mL; IL-13: 18.09 ± 4.07 vs. 24.97 ± 4.44 vs. 31.46 ± 5.22 pg/mL; all *p* < 0.001). The markers of airway remodeling and angiogenesis, including VEGF-A, SDF-1α, TGF-β1, MMP-9, and hyaluronan, also increased significantly with asthma severity (all *p* < 0.001). Notably, serum soluble LYVE-1 (sLYVE-1) levels progressively increased from mild (532.71 ± 74.80 ng/mL) to moderate (621.96 ± 80.72 ng/mL) and severe asthma (687.75 ± 92.86 ng/mL; *p* < 0.001) (Table 2), indicating a potential association between LYVE-1 and disease severity, airway inflammation, and airway remodeling.

**TABLE 2:** Characteristics of the asthma patients with different disease severity.

Variables	Mild group (n = 41)	Moderate group (n = 26)	Severe group (n = 19)	p-value
Age (years)	57.66 ± 8.64	58.77 ± 9.11	60.62 ± 10.39	0.590
Sex (male, %)	21 (51.2%)	16 (61.5%)	13 (68.4%)	0.416
BMI (kg/m^2^)	22.52 ± 2.87	21.82 ± 2.83	21.39 ± 2.27	0.296
Smoking (%)	12 (29.3%)	12 (46.2%)	11 (57.9%)	0.088
FEV1 (L)	2.12 ± 0.54	1.76 ± 0.50	1.55 ± 0.46	<0.001
FEV1/FVC (%)	67.73 ± 4.78	64.62 ± 4.17	62.25 ± 3.88	<0.001
FEV1 predicted (%)	62.42 ± 7.52	57.77 ± 7.30	52.15 ± 7.15	<0.001
Total IgE (IU/mL)	179.27 ± 48.96	206.78 ± 55.15	234.15 ± 62.21	0.002
Eosinophil rate (%)	3.27 ± 0.65	3.81 ± 0.71	4.33 ± 0.77	<0.001
Neutrophil rate (%)	60.12 ± 10.72	61.29 ± 10.76	63.29 ± 10.19	0.560
Th2 (%)	2.69 ± 0.50	3.12 ± 0.53	3.54 ± 0.65	<0.001
Th17 (%)	2.28 ± 0.37	2.71 ± 0.43	3.25 ± 0.47	<0.001
FeNO (ppb)	40.44 ± 5.01	45.67 ± 5.82	52.40 ± 6.08	<0.001
IL-4 (pg/mL)	28.69 ± 3.92	33.36 ± 4.35	38.37 ± 4.91	<0.001
IL-5 (pg/mL)	83.31 ± 11.66	93.23 ± 12.69	101.08 ± 15.82	<0.001
IL-13 (pg/mL)	18.09 ± 4.07	24.97 ± 4.44	31.46 ± 5.22	<0.001
VEGF-A (pg/mL)	106.30 ± 19.72	115.58 ± 21.30	136.97 ± 30.94	<0.001
SDF-1α (pg/mL)	529.47 ± 74.28	578.56 ± 80.53	626.94 ± 96.42	<0.001
TGF-β1 (pg/mL)	42.53 ± 5.28	51.84 ± 6.20	58.15 ± 6.89	<0.001
MMP-9 (ng/mL)	115.32 ± 18.93	124.11 ± 21.23	132.88 ± 20.35	0.007
Hyaluronan (ng/mL)	74.02 ± 11.25	85.74 ± 13.87	97.94 ± 15.85	<0.001
Serum sLYVE1 (ng/mL)	532.71 ± 74.80	621.96 ± 80.72	687.75 ± 92.86	<0.001

Abbreviation: BMI, body mass index; FEV1, forced expiratory volume in one second; FVC, forced vital capacity; IgE, immune globulin E; Th2, T-helper cell type 2; Th17, T-helper cell type 17; FeNO, exhaled nitric oxide; IL-4, interleukin 4; VEGF-A, vascular endothelial growth factor A; SDF-1α, stromal-derived factor 1α; TGF-β1, transforming growth factor-β1; MMP-9, matrix metalloproteinases-9; sLYVE1, soluble lymphatic vessel endothelial hyaluronan receptor 1.

### 3.2 Serum sLYVE-1 is elevated in asthma and correlates with disease severity and pulmonary function

Serum soluble LYVE-1 (sLYVE-1) levels were measured using ELISA to evaluate their association with asthma and disease severity. As shown in Supplementary Figure S1A, circulating sLYVE-1 concentrations were significantly higher in patients with asthma than in healthy controls and progressively increased from patients in remission to those with acute asthma (*p* < 0.001). Further stratification by disease severity demonstrated a stepwise elevation in sLYVE-1 levels from mild to moderate and severe asthma (*p* < 0.01; Supplementary Figure S1B).

To assess the relationship between sLYVE-1 and pulmonary function, Pearson’s correlation analysis was performed in patients with acute asthma. Serum sLYVE-1 levels were negatively correlated with FEV1, FEV1/FVC ratio, and predicted FEV1 (Supplementary Figure S1C-E), indicating that higher sLYVE-1 concentrations were associated with more severe airflow limitations. Collectively, these findings suggest that elevated circulating sLYVE-1 levels reflect both asthma severity and impaired pulmonary function, supporting its potential utility as a biomarker for asthma disease progression.

### 3.3 Serum sLYVE-1 levels correlate with inflammatory and type 2 immune responses in patients with acute asthma

Pearson’s correlation analysis was performed to investigate the relationship between circulating soluble LYVE-1 (sLYVE-1) levels and airway inflammation in patients with acute asthma. As shown in Supplementary Figure S2A-F, serum sLYVE-1 levels were positively correlated with total IgE, eosinophil percentage, neutrophil percentage, Th2 cell proportion, Th17 cell proportion, and FeNO, indicating that higher sLYVE-1 concentrations were associated with heightened systemic and airway inflammatory activity.

In addition, circulating sLYVE-1 levels showed significant positive correlations with type 2 inflammatory cytokines, including interleukin (IL)-4, IL-5, and IL-13 (Supplementary Figure S2G-I), further supporting a close association between sLYVE-1 and Th2-mediated immune responses in acute asthma. Collectively, these findings suggest that sLYVE-1 reflects both the magnitude of airway inflammation and type 2 immune activation, highlighting its potential utility as a biomarker of the inflammatory burden in acute asthma.

### 3.4 Serum sLYVE-1 levels positively correlate with airway remodeling-related biomarkers in patients with acute asthma

Pearson’s correlation analysis was performed to evaluate the association between circulating LYVE-1 levels and airway remodeling in patients with acute asthma. As shown in Supplementary Figure S3, serum sLYVE-1 levels were positively correlated with VEGF-A (Supplementary Figure S3A), SDF-1α (Supplementary Figure S3B), TGF-β1 (Supplementary Figure S3C), MMP-9 (Supplementary Figure S3D), and hyaluronan (Supplementary Figure S3E). These findings indicate that elevated sLYVE-1 levels reflect enhanced airway remodeling activity and suggest its potential role as a biomarker for structural changes in the asthmatic airways.

### 3.5 PDGF-BB induces LYVE-1 expression in ASMCs and LYVE-1 knockdown efficiency

**FIGURE 2:**
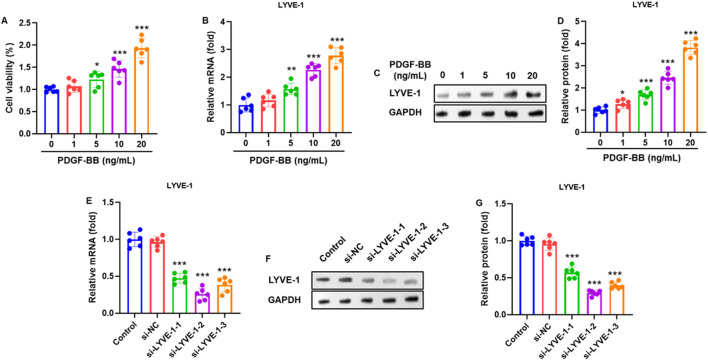
PDGF-BB upregulated LYVE-1 expression in airway smooth muscle cells (ASMCs). **(A)** Human ASMCs were treated with increasing concentrations of PDGF-BB (0, 1, 5, 10, and 20 ng/mL) for 24 h, and cell viability was assessed using the MTT assay. **(B)** LYVE-1 mRNA expression was determined using RT-qPCR. **(C)** Representative western blots showing LYVE-1 protein expression. **(D)** Quantification of LYVE-1 protein levels, normalized to GAPDH. **(E)** ASMCs were transfected with three independent LYVE-1 siRNA sequences (si-LYVE-1#1, #2, and #3) or negative control siRNA (si-NC), and LYVE-1 mRNA expression was assessed using RT-qPCR 48 h after transfection. **(F)** Representative western blots showing LYVE-1 protein expression following siRNA-mediated knockdown. **(G)** Quantification of LYVE-1 protein levels, normalized to GAPDH. Among the three siRNA sequences, si-LYVE-1#2 exhibited the most efficient knockdown and was selected for subsequent experiments. Each dot represents an individual experiment, with bars indicating the mean ± SD of three independent experiments. *p < 0.05, **p < 0.01, ***p < 0.001 vs. control group.

To investigate the regulation of LYVE-1 in airway smooth muscle cells (ASMCs), the cells were treated with increasing concentrations of PDGF-BB (0, 1, 5, 10, and 20 ng/mL) for 24 h. MTT assays demonstrated that PDGF-BB enhanced ASMC viability in a dose-dependent manner (Figure 2A). Correspondingly, LYVE-1 mRNA expression was significantly upregulated following PDGF-BB treatment, as determined by RT-qPCR (Figure 2B). Western blot analysis confirmed a dose-dependent increase in LYVE-1 protein levels (Figure 2C), which were quantified relative to GAPDH (Figure 2D). To assess the functional role of LYVE-1, ASMCs were transfected with three independent LYVE-1 siRNA sequences (si-LYVE-1#1, #2, and #3) or a control siRNA (si-NC). RT-qPCR analysis at 48 h post-transfection revealed a significant knockdown of LYVE-1 mRNA, with si-LYVE-1#2 exhibiting the most potent inhibitory effect (Figure 2E). Western blot analysis corroborated these findings at the protein level (Figure 2F), and quantification confirmed maximal LYVE-1 suppression with the sequence #2 sequence (Figure 2G). Therefore, si-LYVE-1#2 was selected for subsequent functional experiments.

### 3.6 LYVE-1 knockdown inhibits PI3K/Akt signaling and suppresses PDGF-BB-induced ASMC proliferation, migration, and extracellular matrix expression

**FIGURE 3:**
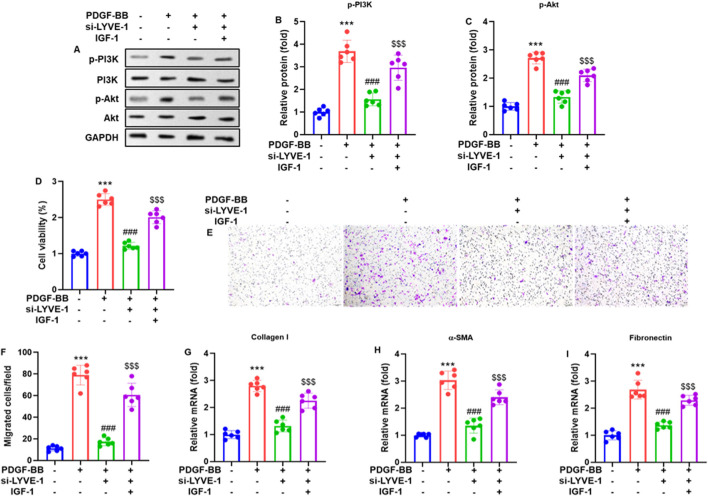
LYVE-1 knockdown inhibited PI3K/Akt signaling, proliferation, migration, and extracellular matrix expression in PDGF-BB-treated ASMCs. ASMCs were transfected with si-LYVE-1 for 24 h and subsequently treated with PDGF-BB (20 ng/mL) in the presence or absence of the PI3K/Akt activator IGF-1 (100 ng/mL) for an additional 24 h. **(A)** Representative western blots showing phosphorylated PI3K (p-PI3K, p85, Tyr458, normalized to total PI3K) and phosphorylated Akt (p-Akt, Ser473, normalized to total Akt). **(B,C)** Quantification of p-PI3K and p-Akt protein levels. **(D)** ASMC viability was assessed using the MTT assay. **(E)** Migration ability of ASMCs determined by Transwell assay and crystal violet staining (0.1%, magnification ×100). **(F)** Quantification of the migrated cells. **(G–I)** RT-qPCR analysis of extracellular matrix gene expression: Collagen I **(G)**, α-SMA **(H)**, and fibronectin **(I)**. Each dot represents an individual experiment, with bars indicating the mean ± SD of three independent experiments. ***p < 0.001 vs. control group; ###*p* < 0.001 vs. PDGF-BB group; $$$ *p* < 0.001 vs. PDGF-BB + si-LYVE-1 group.

To investigate the role of LYVE-1 in PI3K/Akt pathway activation, ASMCs were transfected with LYVE-1 siRNA (si-LYVE-1#2) for 24 h and subsequently treated with PDGF-BB (20 ng/mL) in the presence or absence of the PI3K/Akt activator IGF-1 (100 ng/mL) for another 24 h. Western blot analysis revealed that PDGF-BB treatment markedly increased the phosphorylation of PI3K (p85, Tyr458) and Akt (Ser473) compared that to in the untreated controls. LYVE-1 knockdown significantly attenuated PDGF-BB-induced phosphorylation of both PI3K and Akt (Figures 3A–C). Notably, co-treatment with IGF-1 partially restored PI3K/Akt phosphorylation in LYVE-1-silenced ASMCs, confirming that LYVE-1 modulates PDGF-BB-induced PI3K/Akt pathway activation in airway smooth muscle cells.

MTT assays demonstrated that LYVE-1 silencing significantly reduced PDGF-BB-induced ASMC viability compared to that in the control siRNA (Figure 3D). Transwell migration assays revealed that LYVE-1 knockdown markedly inhibited PDGF-BB-stimulated ASMC migration, as visualized by crystal violet staining (Figure 3E) and quantification of the number of migrated cells (Figure 3F). Additionally, RT-qPCR analysis showed that LYVE-1 silencing significantly decreased the mRNA expression of ECM-related genes, including Collagen I (Figure 3G), α-SMA (Figure 3H), and fibronectin (Figure 3I), indicating that LYVE-1 contributes to PDGF-BB-induced ASMC proliferation, motility, and ECM production.

### 3.7 LYVE-1 knockdown attenuates PDGF-BB-induced inflammatory responses in ASMCs

**FIGURE 4:**
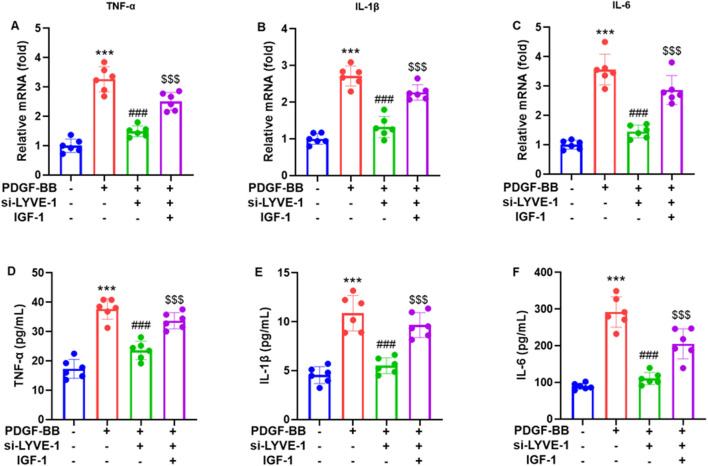
LYVE-1 knockdown suppresses PDGF-BB-stimulated inflammation. RT-qPCR was used to assess mRNA expression the mRNA expression of pro-inflammatory cytokines **(A)** TNF-α, **(B)** IL-1β and **(C)** IL-6 in ASMCs stimulated by PDGF-BB. ELISA was used to measure the concentrations of **(D)** TNF-α, **(E)** IL-1β and **(F)** IL-6 in ASMCs supernatants. Each dot represents an individual experiment, with bars indicating the mean ± SD of three independent experiments. ***p < 0.001 vs. control group; ###*p* < 0.001 vs. PDGF-BB group; $$$ *p* < 0.001 vs. PDGF-BB + si-LYVE-1 group.

To examine the role of LYVE-1 in airway smooth muscle cell (ASMC) inflammation, the cells were transfected with LYVE-1 siRNA (si-LYVE-1#2) and stimulated with PDGF-BB (20 ng/mL). RT-qPCR analysis revealed that LYVE-1 silencing significantly reduced PDGF-BB-induced mRNA expression of the pro-inflammatory cytokines TNF-α (Figure 4A), IL-1β (Figure 4B), and IL-6 (Figure 4C). Consistently, ELISA measurements of ASMC supernatants showed that LYVE-1 knockdown markedly decreased TNF-α (Figure 4D), IL-1β (Figure 4E), and IL-6 (Figure 4F) secretion compared with that in the control siRNA group. These results indicate that LYVE-1 contributes to PDGF-BB-stimulated inflammatory activation in ASMCs, highlighting its role in airway inflammation.

### 3.8 LYVE-1 knockdown reduces intracellular ROS generation and oxidative stress in PDGF-BB-treated ASMCs

**FIGURE 5:**
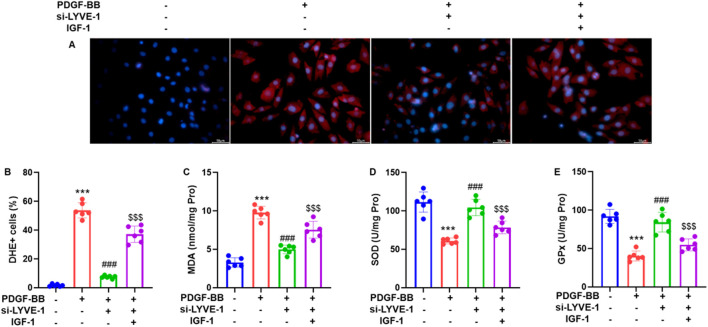
LYVE-1 knockdown inhibits intracellular ROS generation and oxidative stress in PDGF-BB-treated ASMCs. **(A)** Intracellular ROS of ASMCs were assessed by immunofluorescence and DHE staining, and DAPI staining showed cell nucleus (200×). The representative image are shown. **(B)** Quantitative analysis of the percentage of DHE positive cells relative to DAPI + cells. The oxidative stress was evaluated by detecting **(C)** MDA content, **(D)** SOD activity and **(E)** GPx activity in cell lysate. Each dot represents an individual experiment, with bars indicating the mean ± SD of three independent experiments. ***p < 0.001 vs. control group; ###*p* < 0.001 vs. PDGF-BB group; $$$ *p* < 0.001 vs. PDGF-BB + si-LYVE-1 group.

To evaluate the effect of LYVE-1 on oxidative stress, ASMCs were transfected with LYVE-1 siRNA (si-LYVE-1#2) and stimulated with PDGF-BB (20 ng/mL). Immunofluorescence analysis using DHE staining demonstrated that PDGF-BB markedly increased intracellular ROS levels, whereas LYVE-1 knockdown significantly reduced the number of DHE-positive cells relative to DAPI-stained nuclei (Figures 5A,B). Consistently, the assessment of oxidative stress markers in cell lysates showed that LYVE-1 silencing significantly decreased MDA levels (Figure 5C) and increased antioxidant enzyme activities, including SOD (Figure 5D) and GPx (Figure 5E), compared with the control siRNA. These results indicate that LYVE-1 contributes to PDGF-BB-induced oxidative stress in ASMCs and that its knockdown mitigates ROS accumulation and restores the antioxidant defenses.

## 4 Discussion

In the present study, we demonstrated that circulating soluble LYVE-1 (sLYVE-1) levels were elevated in patients with asthma, progressively increase with disease severity, and are inversely associated with pulmonary function. Complementary *in vitro* experiments further revealed that PDGF-BB induced LYVE-1 expression in ASMCs and that LYVE-1 knockdown attenuated PDGF-BB-induced proliferation, migration, oxidative stress, and inflammatory responses by suppressing the PI3K/Akt pathway. Collectively, these findings identify LYVE-1 as a potential circulating biomarker and functional mediator of asthma pathophysiology.

Clinically, serum sLYVE-1 levels were significantly higher in patients with acute asthma than in those in remission and healthy controls and increased incrementally with disease severity. Notably, sLYVE-1 levels were inversely correlated with lung function indices, including FEV1, FEV1/FVC, and predicted FEV1, supporting its relevance as a surrogate marker of airway obstruction and disease progression. Furthermore, sLYVE-1 showed robust positive correlations with key type 2 inflammatory markers, including eosinophil counts, FeNO, and the cytokines IL-4, IL-5, and IL-13, embedding LYVE-1 within the Th2-high asthma endotype (Lambrecht and Hammad, 2015; Fahy, 2015). This observation extends the functional relevance of LYVE-1 beyond lymphatic endothelial biology to the core inflammatory pathways involved in asthma.

The presence of soluble LYVE-1 in circulation is most plausibly explained by the proteolytic shedding of membrane-bound LYVE-1 under inflammatory conditions. Previous studies have demonstrated that LYVE-1 undergoes ectodomain shedding mediated by metalloproteinases, including ADAM17 and membrane-type MMPs, particularly in chronic inflammatory settings (Nishida-Fukuda et al., 2016; Dollt et al., 2017). In asthma, persistent airway inflammation and oxidative stress are well-established activators of matrix metalloproteinases, especially MMP-9, and other membrane-associated sheddases capable of cleaving the extracellular domains of transmembrane receptors. Consistent with this, elevated MMP-9 expression and activity have been reported in the airway tissues and systemic circulation of patients with asthma and are closely linked to inflammatory signaling and tissue remodeling driven by oxidative stress (Belleguic et al., 2002; Kim et al., 2024). Supporting this mechanism, we observed strong positive correlations between sLYVE-1 levels and MMP-9, proinflammatory cytokines, and oxidative stress–related markers. In addition, inflammatory activation and remodeling of the lymphatic endothelium in asthmatic airways may further enhance LYVE-1 expression, turnover, and shedding, thereby contributing to elevated circulating sLYVE-1 concentrations, in line with the increased LYVE-1 dynamics reported in chronic inflammatory diseases (Nishida-Fukuda et al., 2016; Dai et al., 2019).

Importantly, sLYVE-1 levels are strongly associated with mediators central to airway remodeling and angiogenesis, including VEGF-A, SDF-1α, TGF-β1, MMP-9, and hyaluronan (Jakiela et al., 2025; Wilson and Kotsimbos, 2003; Garantziotis et al., 2016; Xu et al., 2025). These correlations suggest a close relationship between sLYVE-1 and structural remodeling processes, such as smooth muscle hypertrophy, extracellular matrix deposition, and neovascularization, which are hallmarks of chronic asthma. This clinical evidence provides a strong rationale for our mechanistic studies in ASMCs.

At the cellular level, PDGF-BB induced a dose-dependent increase in LYVE-1 expression in ASMCs. Silencing LYVE-1 markedly suppressed PDGF-BB-induced activation of the PI3K/Akt pathway, which is a central regulator of ASMC proliferation, migration, and extracellular matrix synthesis (Yu et al., 2016; Xu et al., 2025). Consequently, LYVE-1 knockdown reduced ASMC viability, migratory capacity, and expression of remodeling-associated genes, including collagen I, α-smooth muscle actin, and fibronectin, indicating that LYVE-1 functions as a critical signaling mediator linking growth factor stimulation to airway remodeling.

In addition to its role in structural remodeling, LYVE-1 contributed to oxidative stress and inflammatory signaling in ASMCs. LYVE-1 silencing reduced reactive oxygen species and malondialdehyde levels while enhancing antioxidant enzyme activities, underscoring its role in redox imbalance. Given that oxidative stress amplifies airway inflammation and remodeling in asthma, this finding has particular pharmacological relevance (Comhair and Erzurum, 2010; Riedl and Nel, 2008). Consistently, LYVE-1 knockdown suppressed PDGF-BB-induced secretion of TNF-α, IL-1β, and IL-6, suggesting that LYVE-1 participates in a feed-forward loop linking oxidative stress and inflammation.

Mechanistically, our findings suggest that LYVE-1, traditionally regarded as a lymphatic hyaluronan receptor, is functionally repurposed in ASMCs to integrate inflammatory, oxidative, and remodeling signals via PI3K/Akt-dependent pathways. This concept aligns with emerging evidence that LYVE-1-expressing cells regulate smooth muscle mechanics and vascular remodeling via hyaluronan–MMP-9 interactions (Lim et al., 2018; Elfstrum et al., 2024). Extending these observations to the airway, LYVE-1 may represent a novel therapeutic target at the intersection of inflammation and airway remodeling in patients with asthma.

This study has several limitations. First, it was a cross-sectional study, and longitudinal data are needed to assess whether circulating sLYVE-1 predicts future airway remodeling, lung function decline, or response to therapy. Second, although our *in vitro* experiments demonstrated the ASMC-intrinsic effects of LYVE-1, *in vivo* validation using animal models or human bronchial tissues is required to confirm the relevance of these findings in the airway environment. Third, the upstream mechanisms driving LYVE-1 induction in ASMCs, such as the specific transcriptional regulators activated by PDGF-BB, remain to be elucidated. Finally, the clinical utility of sLYVE-1 as a biomarker requires further evaluation of its sensitivity, specificity, and therapeutic responsiveness.

Future studies should address these limitations by performing longitudinal analyses to determine the predictive value of sLYVE-1 in disease progression and therapeutic responses. *In vivo* experiments are warranted to validate LYVE-1’s functional role in airway smooth muscle remodeling. Moreover, investigating the upstream signaling pathways and transcriptional regulators that mediate LYVE-1 induction may uncover novel therapeutic targets. Large-scale clinical studies are needed to establish the potential of sLYVE-1 as a reliable biomarker for asthma severity and inflammatory activity.

## 5 Conclusion

**FIGURE 6:**
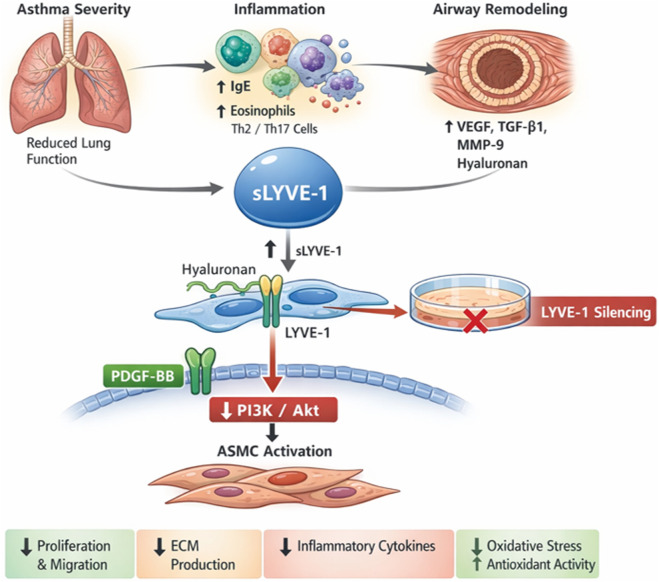
Schematic illustration of the proposed role of LYVE-1 in asthma-associated airway inflammation and remodeling. Circulating soluble LYVE-1 (sLYVE-1) levels are elevated in patients with asthma and increase with disease severity, correlating with impaired lung function, enhanced type 2/Th17 immune responses, and increased airway remodeling–related mediators. In airway smooth muscle cells (ASMCs), platelet-derived growth factor-BB (PDGF-BB) induces LYVE-1 expression, which promotes the activation of the PI3K/Akt signaling pathway. Activation of PI3K/Akt drives ASMC proliferation and migration, extracellular matrix (ECM) production, proinflammatory cytokine release, and oxidative stress, thereby contributing to airway remodeling. LYVE-1 silencing suppressed PI3K/Akt phosphorylation and attenuated PDGF-BB-induced ASMC activation, inflammation, and oxidative stress, while enhancing antioxidant activity. Together, these findings identify LYVE-1 as a key mediator linking airway inflammation to structural remodeling in asthma and highlight its potential as a biomarker and therapeutic target for asthma.

Our study demonstrates that LYVE-1 serves as both a biomarker and a mechanistic mediator in asthma. Circulating soluble LYVE-1 (sLYVE-1) levels reflect disease severity, airway inflammation, and remodeling, and correlate with pulmonary function decline and type 2 immune activation. Mechanistically, LYVE-1 promotes PDGF-BB-induced ASMC proliferation, migration, extracellular matrix production, inflammatory cytokine release, and oxidative stress through the activation of the PI3K/Akt pathway (Figure 6). These findings highlight LYVE-1 as a potential therapeutic target, suggesting that its inhibition may attenuate ASMC dysfunction and airway remodeling. Future *in vivo* studies are warranted to assess whether targeting LYVE-1 can reverse structural airway changes and improve lung function in patients with asthma.

## Data Availability

The datasets presented in this study can be found in online repositories. The names of the repository/repositories and accession number(s) can be found in the article/Supplementary Material.
