# Supplementary figures and images for: LYVE-1 identifies asthma and drives PDGF-BB-induced proliferation, migration, and oxidative stress in airway smooth muscle cells via the PI3K/Akt pathway
Source: Front Pharmacol. 2026 Feb 11;17:1738301. doi: 10.3389/fphar.2026.1738301 (PMC12932558; doi:10.3389/fphar.2026.1738301)

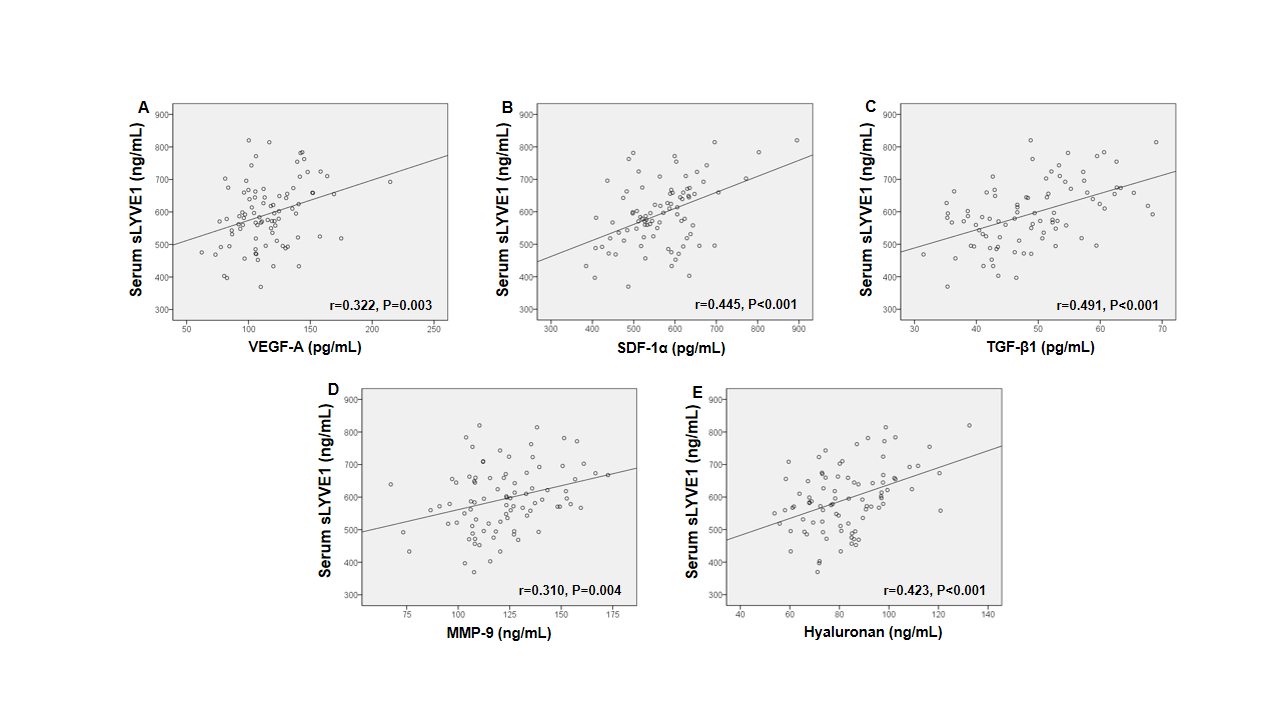

Supplement: Supplementary file 1 [file Image3.TIF]

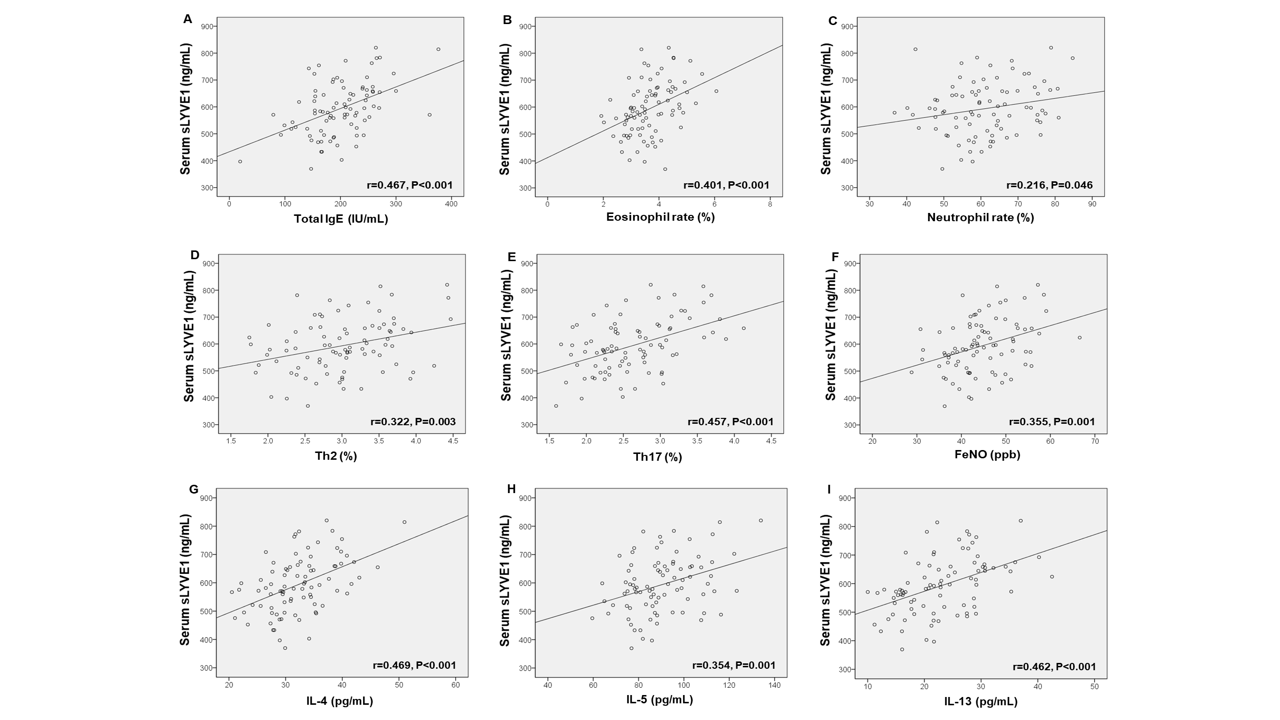

Supplement: Supplementary file 2 [file Image2.TIF]

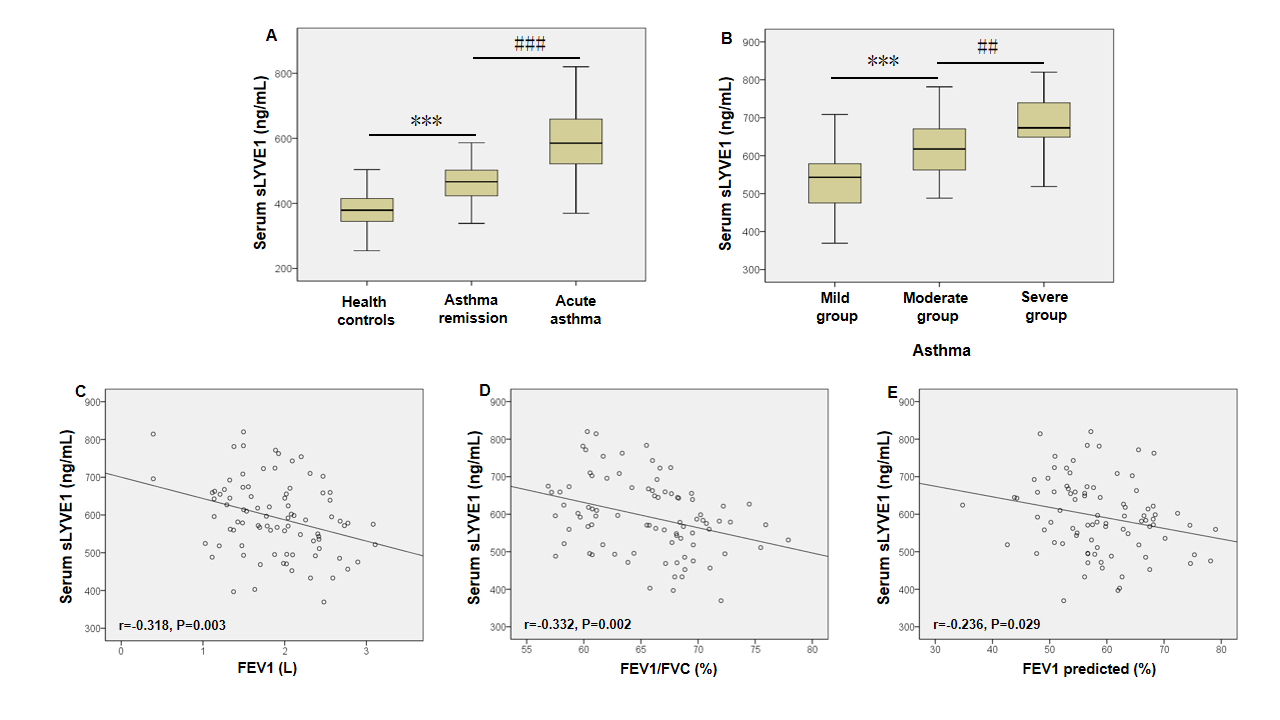

Supplement: Supplementary file 3 [file Image1.TIF]
