# Supplementary material for: LYVE-1 identifies asthma and drives PDGF-BB-induced proliferation, migration, and oxidative stress in airway smooth muscle cells via the PI3K/Akt pathway
Source: Front Pharmacol. 2026 Feb 11;17:1738301. doi: 10.3389/fphar.2026.1738301 (PMC12932558; doi:10.3389/fphar.2026.1738301)
Supplement: Supplementary file 4 [file Table1.DOC]

**Supplementary Table 1.** List of oligonucleotide primer sequences used in the investigation.

| Genes | Forward primer (5′-3′) | Reverse primer (5′-3′) |
| --- | --- | --- |
| Human LYVE1 | GCCGACAGTTTGCAGCCTATTG | CCGAGTAGGTACTGTCACTGAC |
| Human TNF-α | CTGGGCAGGTCTACTTTGGG | CTGGAGGCCCCAGTTTGAAT |
| Human IL-1β | CCAGGGACAGGATATGGAGCA | TTCAACACGCAGGACAGGTACAG |
| Human IL-6 | TCCACAAGCGCCTTCGGTC | GGTCAGGGGTGGTTATTGCAT |
| Human Collagen I | GAGGGCCAAGACGAAGACATC | CAGATCACGTCATCGCACAAC |
| Human α-SMA | AAAGCAAGTCCTCCAGCGTT | TTAGTCCCGGGGATAGGCAA |
| Human Fibronectin | GATACCATCATCCCAGCTGTTC | CAGGAAGTTGGTTAAATCAATGGA |
| Human GAPDH | CATGTTGCAACCGGGAAGGA | CGCCCAATACGACCAAATCAG |
